# Gene-Specific Function Prediction for Non-Synonymous Mutations in Monogenic Diabetes Genes

**DOI:** 10.1371/journal.pone.0104452

**Published:** 2014-08-19

**Authors:** Quan Li, Xiaoming Liu, Richard A. Gibbs, Eric Boerwinkle, Constantin Polychronakos, Hui-Qi Qu

**Affiliations:** 1 Endocrine Genetics Lab, The McGill University Health Center (Montreal Children's Hospital), Montréal, Québec, Canada; 2 Human Genetics Center, Division of Epidemiology, Human Genetics and Environmental Sciences, The University of Texas School of Public Health, Houston, Texas, United States of America; 3 Human Genome Sequencing Center, Baylor College of Medicine, Houston, Texas, United States of America; 4 Department of Molecular and Human Genetics, Baylor College of Medicine, Houston, Texas, United States of America; Odense University hospital, Denmark

## Abstract

The rapid progress of genomic technologies has been providing new opportunities to address the need of maturity-onset diabetes of the young (MODY) molecular diagnosis. However, whether a new mutation causes MODY can be questionable. A number of *in silico* methods have been developed to predict functional effects of rare human mutations. The purpose of this study is to compare the performance of different bioinformatics methods in the functional prediction of nonsynonymous mutations in each MODY gene, and provides reference matrices to assist the molecular diagnosis of MODY. Our study showed that the prediction scores by different methods of the diabetes mutations were highly correlated, but were more complimentary than replacement to each other. The available *in silico* methods for the prediction of diabetes mutations had varied performances across different genes. Applying gene-specific thresholds defined by this study may be able to increase the performance of *in silico* prediction of disease-causing mutations.

## Introduction

To date, a number of methods have been developed to predict functional effects of rare human mutations based on the impact of protein function and/or evolutionary conservation [1]–[3]. These methods are valuable to assist the diagnosis of monogenic inheritance diseases. In the area of diabetes, there is a common monogenic form, i.e. maturity-onset diabetes of the young (MODY). MODY accounts for ∼1% to 5% of all cases of diabetes, while it is mainly seen in young adults (≤25 years old) [4]. As an autosomal dominant inherited form of diabetes, MODY is caused by gene mutations leading to insufficient insulin production without or with minimal insulin resistance [5]. To date, at least 13 genes have been identified with mutations that cause MODY, i.e. *HNF4A* (MODY1) [6], *GCK* (MODY2) [7], *HNF1A* (MODY3) [6], *PDX1* (MODY4) [8], *HNF1B* (MODY5) [9], *NEUROD1* (MODY6) [10], *KLF11* (MODY7) [11], *CEL* (MODY8) [12], *PAX4* (MODY9) [13], *INS* (MODY10) [14], *BLK* (MODY11) [15], *ABCC8* (MODY12) [16], [17], and *KCNJ11* (MODY13) [16], [17].

MODY caused by different gene mutations may have different severities of diabetes and different drug responses to diabetes medications [18]. For example, MODY2 (accounts for ∼20% of all MODY cases [19]) caused by *GCK* gene mutations tends to have mild hyperglycaemia without obvious glycosuria. Patients with MODY2 are often asymptomatic [20] or only identified in women during pregnancy and diagnosed as gestational diabetes [21]. Most patients with MODY2 can have blood glucose satisfactorily controlled by diet therapy and don't need hypoglycemic medication [22], [23]. In contrast, MODY3 caused by *HNF1A* mutations, the most common type of MODY that accounts for ∼63% of all MODY cases [19], tends to have obvious glycosuria because of impaired glucose-stimulated insulin secretion [24], as well as decreased renal threshold for glucose [25]. MODY3 patients tend to have good response to sulphonylurea treatment and don't rely on insulin therapy [18], [26]. Because of the implications of pharcogenetics and personalized medicine, molecular diagnosis of MODY has clinical importance for clinical decision and for genetic counseling [18], [26]. However, because of unavailability and expense of MODY molecular diagnosis, it is not uncommon that MODY patients are classified as type 2 diabetes [27], [28] and occasionally as type 1 [29].

The rapid progress of advanced genomic technologies has been providing new opportunities to address the need of MODY molecular diagnosis. The identification of mutations in MODY genes by sequencing technologies will enable the molecular diagnosis of MODY, whereas a new issue is emerging. Most mutations causing MODY are nonsynonymous single-nucleotide mutations causing the change of an amino acid residue (according to The Human Gene Mutation Database [30], http://www.hgmd.org/). High throughput sequencing technologies enable screening of a large number of patients and parallel sequencing of a large number of genes. If a known MODY gene mutation is identified in a patient suspected of MODY, the molecular diagnosis of MODY can be established. However, the increased throughput of sequencing technologies is likely to produce increased numbers of missense variants whose causative role in MODY can be questionable. Bioinformatics tools, e.g. SIFT (http://sift.jcvi.org/) [31] and PolyPhen (http://genetics.bwh.harvard.edu/pph2/index.shtml) [32], are often used to assess the pathogenicity of a nonsynonymous mutation [27]. Because the limitations of *in silico* methods, the functional prediction of a nonsynonymous mutation lacks a gold standard. To date, a number of bioinformatics methods besides SIFT and PolyPhen, based on different algorithms, have been developed [1]–[3]. The purpose of this study is to compare the performances of different bioinformatics methods in the functional prediction of nonsynonymous mutations in each MODY gene, and to provide reference matrices to assist the molecular diagnosis of MODY.

## Methods

### Data source

The diabetes mutation data analyzed in this study were acquired from the Human Gene Mutation Database (HGMD) 2013.4 release (http://www.hgmd.org/) [30]. As the purpose of this study is to assess the prediction performances of different *in silico* methods for nonsynonymous single-nucleotide mutations, insertion/deletion mutations (InDels) are not included in this study. Altogether, 1,130 nonsynonymous single-nucleotide mutations from 24 genes have been reported causing MODY or neonatal diabetes. Among these genes, 7 genes harbor more than 30 single-nucleotide mutations within each gene with the total of 1,091 diabetes mutations (Table 1), while the other 17 genes harbor ≤6 diabetes mutations in each gene. To enable statistical comparisons of different *in silico* methods across different genes, those 17 genes with ≤6 diabetes mutations were not involved in this study. Among the 1,091 mutations, 155 mutations from the genes *ABCC8*, *GCK*, *INS*, or *KCNJ11*, have the phenotype of neonatal diabetes, either transient or permanent. The other 936 mutations in the 7 genes have the phenotype of MODY.

**Table 1:** MODY or neonatal diabetes genes and mutations (n = 1091).

Gene symbol	Diabetic mutations* (n)	Control mutations (n)	Chromosome position	Gene name
*HNF4A*	86	57	20q12-q13.1	hepatocyte nuclear factor 4, alpha
*GCK*	479	22	7p15.3-p15.1	glucokinase (hexokinase 4, maturity onset diabetes of the young 2)
*HNF1A*	324	78	12q24.2	hepatocyte nuclear factor-1 (HNF1) homeobox A
*HNF1B*	36	52	17cen-q21.3	hepatocyte nuclear factor-1 (HNF1) homeobox B
*INS*	41	14	11p15.5	insulin
*ABCC8*	64	185	11p15.1	ATP-binding cassette, sub-family C (CFTR/MRP), member 8
*KCNJ11*	61	65	11p15.1	potassium inwardly-rectifying channel, subfamily J, member 11

* Number of diabetes-causing single nucleotide mutations recorded in the Human Gene Mutation Database (HGMD) 2013.4 release (http://www.hgmd.org/) [30].

Control nonsynonymous single-nucleotide mutations in the diabetes genes were acquired from the NHLBI GO Exome Sequencing Project (ESP) [33], [34], the ARIC samples [35] in the CHARGE Exome Sequencing Project [36], and the 1000 Genome Project [37], excluding mutations recorded in the HGMD database.

### Functional prediction of nonsynonymous single-nucleotide mutations

Eleven methods, including PhyloP [38], GERP++ RS [39], SiPhy [40], SIFT [31], PolyPhen-2 [32], the likelihood ratio test (LRT) [41], MutationTaster [42], Mutation Assessor [43], FATHMM [44], RadialSVM score [3], and logistic regression (LR) score [3], were covered in the dbNSFP database [45], [46] and compared in this study (Table 2). Among the 1,091 mutations involved in this analysis, 104 mutations from the genes *GCK*, *HNF1A*, *HNF1B*, *HNF4A*, and *INS*, are nonsense mutations, i.e. producing a pre-termination codon; two other mutations from the gene *GCK* replace a termination codon with an amino acid codon. For these mutations, the methods, PolyPhen-2 HDIV, PolyPhen-2 HVAR, MutationAssessor, FATHMM, Radial SVM score, LR score are not applicable to nonsense mutations or mutations assumed with highly damaging potential. Other methods, except MutationTaster, tend to have higher error rates (false negative rates, FNR), compared to the prediction of amino acid substitution mutations, i.e. SIFT FNR = 72%, GERP++ RS FNR = 40%, PhyloP FNR = 35%, SiPhy FNR = 26%, LRT FNR = 21%, and MutationTaster FNR = 3%, for the prediction of nonsense mutations. Compared with amino acid substitutions, the assessment of the functional effect of nonsense mutations tends to be less of an issue. The final analysis of this study involved 985 nonsynonymous single-nucleotide mutations. The quantitative performances of these methods were compared by the Spearman's rank correlation test [47] and the ANOVA test using the IBM SPSS Statistics 19 software ((IBM SPSS Inc., Chicago, IL, USA). To re-define gene-specific thresholds of deleterious mutations, the receiver operator characteristic (ROC) analysis was calculated by the sensitivity and specificity values of screening series of cutoffs of each method for each gene. A redefined threshold was identified using the maximum Matthews correlation coefficient (MCC) [48].

**Table 2:** Methods for function prediction for non-synonymous mutations*.

Method	Deleterious Threshold	Algorithm
PhyloP	>1.6	PhyloP calculates basewise conservation score from Multiz alignment [55] of 46 vertebrate species (ftp://ccg.vital-it.ch/mga/hg19/phylop/phylop.html) [38].
GERP++ RS	>4.4	GERP++ RS calculates site-specific “rejected substitutions” (RS) scores and to discover evolutionarily constrained elements based on maximum likelihood evolutionary rate estimation [39].
SiPhy	>12.17	SiPhy detects bases under selection from a multiple alignment data using a hidden Markov model. (http://www.broadinstitute.org/genome_bio/siphy/) [40].
SIFT	>0.95	SIFT prediction is based on the degree of conservation of amino acid residues in sequence alignments derived from closely related sequences, collected through PSI-BLAST.(http://sift.jcvi.org/) [31].
PolyPhen-2	>0.5	PolyPhen-2 (Polymorphism Phenotyping v2) predicts the functional significance of an amino acid substitution by Naïve Bayes classifier, using sequence-based and structure-based predictive features. HDIV, or HumDiv, identifies human damaging mutations by assuming differences between human proteins and their closely related mammalian homologs as non-damaging. HVAR, or HumVar, identifies human disease-causing mutations by assuming common human nsSNPs as non-damaging. (http://genetics.bwh.harvard.edu/pph2/index.shtml) [32].
LRT	>0.999	The likelihood ratio test (LRT) identifies conserved amino acid positions and deleterious mutations using a comparative genomics data set of multiple vertebrate species. (http://www.genetics.wustl.edu/jflab/lrt_query.html) [41].
MutationTaster	>0.5	MutationTaster evaluates the disease-causing potential of DNA sequence alterations by Naïve Bayes classifier, integrating information Of evolutionary conservation, splice-site changes, loss of protein features and changes that might affect the amount of mRNA from different biomedical databases and uses established analysis tools. (http://www.mutationtaster.org/) [42].
Mutation Assessor	>0.65	Mutation Assessor predicts the functional impact of amino-acid substitutions in proteins based on evolutionary conservation of the affected amino acid in protein homologs. (http://mutationassessor.org) [43].
FATHMM	>0.453	The Functional Analysis through Hidden Markov Models (FATHMM) Predicts the functional consequences of cancer-associated amino acid substitutions using a model weighted for inherited disease mutations (http://fathmm.biocompute.org.uk) [44].
RadialSVM score	>0.5	RadialSVM score is an ensemble-based approach integrating multiple scoring systems (function prediction and conservation Score) by radial support vector machine (SVM) [3].
LR score	>0.5	LR score is an ensemble-based approach integrating multiple scoring systems (function prediction and conservation Score) by logistic regression (LR) [3].

* Extensively comparisons of these methods genome-widely have been studied by Thusberg et al. [1], [2] and Dong et al. [3]. Deleterious thresholds for PhyloP, GERP++ RS and SiPhy are defined according to the study by Dong et al. [3]. Deleterious thresholds for SIFT, LRT, MutationTaster, Mutation Assessor, FATHMM and RAdialSVM are based on converted scores used in dbNSFP version 2.3 [45], [46] (converted score is designated as S_c_ and original score is designated as S_o_): SIFT: S_c_ = 1-S_o_; LRT: S_c_ = 1- S_o_ *0.5 if Ω<1, or S_c_ = S_o_ *0.5 if Ω> = 1; MutationTaster: S_c_ = S_o_ if the prediction is “A” or “D” or S_c_ = 1- S_o_ if the prediction is “N” or “P”; Mutation Assessor: S_c_ = (S_o_ -(−5.545))/(5.975-(−5.545)); FATHMM: S_c_ = 1-(S_o_ -(−16.13))/(10.64-(−16.13)); RadialSVM: S_c_ = (1+ S_o_/3.03993691875303)*0.5 if predicted “D” and S_c_ = (1- S_o_/-2.00575697514507)*0.5 otherwise. More details of the conversion can be found at http://dbnsfp.houstonbioinformatics.org/dbNSFPzip/dbNSFP2.3.readme.txt.

## Results and Discussion

In our analysis, the prediction scores by different methods of the diabetes mutations are highly correlated (Table 3). The highest correlations are seen between RadialSVM score and LR score (r = 0.957), PolyPhen-2 HDIV and PolyPhen-2 HVAR (r = 0.89), and phyloP and GERP++ RS (r = 0.871), while the other correlations have r<0.80. Therefore, in spite of the high statistical significance of correlations between different methods, different methods may not be able to replace each other except for the above three pairs. Especially, the FATHMM method has no obvious correlation with PhyloP, GERP++ RS, and LRT, while the correlation with MutationTaster is less significant. On the other hand, we observed significantly varied performances in detecting deleterious mutations by different methods (Table 4). Prediction errors by the *in silico* methods highlight the limitations of these methods and the need for cautious applications of the *in silico* prediction in data explanation. Among different methods, FATHMM has the lowest false negative rate (FNR = 1%), but also the highest false positive rate (FPR = 95%) [Matthews correlation coefficient (MCC) = 0.127]. Considering the lack of correlation of FATHMM with the PhyloP, GERP++ RS, and LRT, caution should be taken when explaining the FATHMM results because of its high FPR and low MCC. The highest MCC scores were seen in the RadialSVM score (MCC = 0.474, FNR = 5%), PolyPhen-2 HDIV (MMC = 0.447, FNR = 9%), PolyPhen-2 HVAR (MCC = 0.434, FNR = 16%) and LR score (MCC = 0.393, FNR = 4%).

**Table 3:** Correlations of different Methods for function prediction for non-synonymous mutations causing diabetes [Spearman's ρ (P value)].

Methods	phyloP	GERP++ RS	SiPhy	SIFT	PolyPhen-2 HDIV	PolyPhen-2 HVAR	LRT	MutationTaster	Mutation Assessor	FATHMM	RadialSVM score
GERP++ RS	0.798 (P = 1.13E-218)										
SiPhy	0.857 (P = 2.42E-285)	0.718 (P = 1.15E-156)									
SIFT	0.018 (P = 5.92E-01)	0.062 (P = 6.75E-02)	0.087 (P = 1.09E-02)								
PolyPhen-2 HDIV	0.228 (P = 1.16E-11)	0.192 (P = 1.20E-08)	0.278 (P = 9.60E-17)	0.506 (P = 8.57E-57)							
PolyPhen-2 HVAR	0.205 (P = 1.27E-09)	0.19 (P = 1.85E-08)	0.233 (P = 3.89E-12)	0.496 (P = 2.10E-54)	0.88 (P = 3.88E-281)						
LRT	0.332 (P = 3.28E-23)	0.412 (P = 5.32E-36)	0.388 (P = 9.10E-32)	0.252 (P = 1.23E-13)	0.38 (P = 2.13E-30)	0.398 (P = 1.58E-33)					
MutationTaster	0.298 (P = 7.40E-21)	0.33 (P = 1.82E-25)	0.339 (P = 6.75E-27)	0.288 (P = 7.50E-18)	0.348 (P = 6.44E-26)	0.354 (P = 6.86E-27)	0.332 (P = 2.93E-23)				
Mutation Assessor	0.089 (P = 8.79E-03)	0.158 (P = 3.00E-06)	0.158 (P = 3.03E-06)	0.633 (P = 3.11E-96)	0.516 (P = 3.30E-59)	0.493 (P = 2.16E-53)	0.409 (P = 2.73E-35)	0.321 (P = 2.58E-22)			
FATHMM	0.071 (P = 3.75E-02)	0.042 (P = 2.13E-01)	0.087 (P = 1.07E-02)	0.27 (P = 1.09E-15)	0.297 (P = 5.62E-19)	0.342 (P = 5.75E-25)	0.126 (P = 2.28E-04)	0.13 (P = 1.32E-04)	0.297 (P = 8.20E-19)		
RadialSVM score	0.188 (P = 2.08E-08)	0.233 (P = 2.51E-12)	0.254 (P = 1.84E-14)	0.492 (P = 2.33E-53)	0.516 (P = 6.04E-60)	0.473 (P = 2.46E-49)	0.268 (P = 2.29E-15)	0.275 (P = 8.71E-17)	0.398 (P = 1.97E-34)	0.409 (P = 4.23E-36)	
LR score	0.21 (P = 3.08E-10)	0.211 (P = 2.74E-10)	0.272 (P = 1.96E-16)	0.548 (P = 1.82E-68)	0.603 (P = 9.85E-87)	0.621 (P = 5.02E-93)	0.344 (P = 5.86E-25)	0.303 (P = 3.77E-20)	0.646 (P = 6.66E-104)	0.852 (P = 5.43E-244)	0.634 (P = 3.56E-100)

**Table 4:** Method comparisons for function prediction for non-synonymous mutations causing diabetes.

Methods	Missing Rate*	False Negative Rate	False Positive Rate**	MCC
PhyloP	0%	18%	53%	0.300
GERP++ RS	0%	21%	52%	0.281
SiPhy	0%	16%	51%	0.342
SIFT	13%	25%	39%	0.350
PolyPhen-2 HDIV	15%	9%	51%	0.447
PolyPhen-2 HVAR	15%	16%	42%	0.434
LRT	18%	7%	68%	0.324
MutationTaster	3%	3%	77%	0.333
Mutation Assessor	15%	30%	32%	0.362
FATHMM	14%	1%	95%	0.127
RadialSVM score	8%	5%	57%	0.474
LR score	8%	4%	69%	0.393

* The missing rate refers to the percentage of mutations that a method is inapplicable;

**The false positive rate was calculated by nonsynonymous single-nucleotide mutations in the diabetes genes acquired from the NHLBI GO Exome Sequencing Project (ESP) [33], the CHARGE Exome Sequencing Project [34], [36], and the 1000 Genome Project [37], excluding mutations recorded in the HGMD database.

Our investigation further disclosed significant differences of the quantitative performances of different methods, except SIFT, across different genes (Table 5). Varied performances across genes highlight another aspect of limitation of these *in silico* methods. The distribution of the prediction scores presented in Table 5 may be able to serve as a matrix to assist the assessment of functional effects of new mutations in these diabetes genes.

**Table 5:** Prediction score comparisons of diabetes mutations in different genes [Mean±Standard Deviation, N (Maximum/Percentile 75/Median/Percentile 25/Minimum)].

Methods	GCK(MODY2)	INS(MODY10)	KCNJ11(MODY13)	ABCC8(MODY12)	HNF1A(MODY3)	HNF1B(MODY5)	HNF4A(MODY1)	ANOVA *P* value	Overall
**PhyloP**	2.120±0.693, 437 (2.941/2.553/2.285/1.981/−0.445)	1.206±0.852, 38 (2.069/1.918/1.585/0.590/−1.023)	2.046±0.444, 61 (2.548/2.398/2.084/1.942/−0.009)	2.219±0.556, 64 (2.941/2.607/2.331/1.990/−0.403)	1.793±0.791, 283 (2.882/2.246/2.045/1.662/−3.003)	2.377±0.652, 28 (2.890/2.882/2.684/2.162/0.768)	1.894±0.841, 74 (2.814/2.481/2.282/1.226/−1.309)	1.02E-17	1.983±0.752, 985 (2.941/2.449/2.167/1.807/−3.003/)
**GERP++ RS**	4.969±1.305, 437 (6.170/5.690/5.220/4.770/−3.060)	2.353±1.724, 38 (4.020/3.580/2.800/1.853/−3.200)	4.807±0.862, 61 (5.430/5.290/5.160/4.570/−0.548)	5.135±0.803, 64 (6.170/5.490/5.310/4.993/0.768)	4.185±2.020, 283 (6.060/4.910/4.650/4.340/−12.100)	5.279±0.773, 28 (6.060/5.810/5.560/5.110/3.230)	4.591±1.862, 74 (5.930/5.400/5.160/4.328/−7.160)	1.55E-25	4.624±1.654, 985 (6.170/5.430/5.050/4.490/−12.100/)
**SiPhy**	16.241±3.196, 437 (20.490/18.618/16.477/14.725/2.501)	9.288±4.002, 38 (14.890/11.956/9.950/7.312/1.486)	15.732±2.899, 61 (19.243/18.636/15.481/13.607/6.945)	16.327±2.609, 64 (20.567/18.577/16.174/14.642/10.313)	13.956±3.649, 283 (19.609/16.972/14.016/12.608/0.949)	16.300±3.457, 28 (19.609/18.716/17.832/14.263/8.432)	15.254±4.259, 74 (20.336/18.810/15.601/12.647/2.815)	2.26E-37	15.218±3.738, 985 (20.567/18.332/15.716/13.607/0.949/)
**SIFT**	0.940±0.151, 383 (1.000/1.000/1.000/0.970/0.000)	0.956±0.140, 32 (1.000/1.000/1.000/1.000/0.360)	0.906±0.172, 52 (1.000/1.000/0.990/0.868/0.210)	0.888±0.204, 59 (1.000/1.000/0.990/0.875/0.000)	0.918±0.184, 242 (1.000/1.000/1.000/0.933/0.000)	0.922±0.205, 27 (1.000/1.000/0.990/0.965/0.000)	0.921±0.168, 62 (1.000/1.000/1.000/0.953/0.250)	0.247	0.927±0.169, 857 (1.000/1.000/1.000/0.950/0.000/)
**PolyPhen-2 HDIV**	0.917±0.229, 389 (1.000/1.000/1.000/0.988/0.000)	0.906±0.290, 32 (1.000/1.000/1.000/0.998/0.009)	0.964±0.151, 52 (1.000/1.000/1.000/0.996/0.004)	0.821±0.330, 58 (1.000/1.000/0.989/0.858/0.001)	0.870±0.269, 242 (1.000/1.000/0.999/0.920/0.000)	0.909±0.252, 27 (1.000/1.000/1.000/0.999/0.000)	0.883±0.276, 63 (1.000/1.000/1.000/0.995/0.019)	0.0247	0.897±0.253, 863 (1.000/1.000/1.000/0.975/0.000/)
**PolyPhen-2 HVAR**	0.850±0.280, 389 (1.000/0.999/0.995/0.880/0.000)	0.893±0.292, 32 (1.000/1.000/1.000/0.973/0.005)	0.925±0.174, 52 (1.000/1.000/0.998/0.957/0.016)	0.704±0.334, 58 (1.000/0.991/0.806/0.530/0.001)	0.781±0.319, 242 (1.000/0.999/0.980/0.589/0.000)	0.882±0.270, 27 (1.000/1.000/0.986/0.974/0.007)	0.821±0.324, 63 (1.000/0.999/0.994/0.896/0.002)	1.88E-04	0.826±0.297, 863 (1.000/0.999/0.992/0.793/0.000/)
**LRT**	0.999±0.005, 385 (1.000/1.000/1.000/1.000/0.936)	0.971±0.137, 23 (1.000/1.000/1.000/1.000/0.341)	0.994±0.046, 52 (1.000/1.000/1.000/1.000/0.668)	1.000±0.003, 58 (1.000/1.000/1.000/1.000/0.975)	0.994±0.029, 239 (1.000/1.000/1.000/1.000/0.704)	1.000±0.000, 27 (1.000/1.000/1.000/1.000/0.998)	0.997±0.012, 62 (1.000/1.000/1.000/1.000/0.948)	5.86E-04	0.997±0.030, 846 (1.000/1.000/1.000/1.000/0.341/)
**MutationTaster**	0.988±0.101, 428 (1.000/1.000/1.000/1.000/0.001)	0.752±0.437, 32 (1.000/1.000/1.000/0.764/0.000)	0.983±0.128, 61 (1.000/1.000/1.000/1.000/0.000)	0.984±0.125, 64 (1.000/1.000/1.000/1.000/0.000)	0.968±0.165, 266 (1.000/1.000/1.000/1.000/0.000)	0.967±0.173, 28 (1.000/1.000/1.000/1.000/0.087)	0.991±0.069, 66 (1.000/1.000/1.000/1.000/0.439)	3.53E-14	0.974±0.153, 945 (1.000/1.000/1.000/1.000/0.000/)
**Mutation Assessor**	0.720±0.086, 388 (0.837/0.796/0.734/0.674/0.442)	0.752±0.077, 30 (0.816/0.805/0.778/0.728/0.553)	0.660±0.085, 61 (0.802/0.715/0.666/0.605/0.464)	0.671±0.083, 64 (0.886/0.703/0.680/0.636/0.356)	0.638±0.054, 237 (0.697/0.679/0.655/0.619/0.434)	0.665±0.047, 27 (0.706/0.697/0.677/0.658/0.547)	0.707±0.106, 62 (0.887/0.788/0.732/0.661/0.434)	6.27E-36	0.688±0.087, 869 (0.887/0.757/0.685/0.641/0.356/)
**FATHMM**	0.587±0.030, 389 (0.683/0.604/0.583/0.560/0.541)	0.531±0.052, 32 (0.640/0.578/0.503/0.492/0.470)	0.531±0.020, 52 (0.563/0.546/0.534/0.522/0.479)	0.512±0.032, 58 (0.575/0.530/0.507/0.497/0.405)	0.577±0.037, 241 (0.685/0.603/0.576/0.546/0.494)	0.582±0.034, 27 (0.626/0.612/0.588/0.544/0.537)	0.542±0.042, 63 (0.612/0.556/0.550/0.537/0.408)	9.03E-73	0.570±0.042, 862 (0.685/0.598/0.571/0.547/0.405/)
**RadialSVM score**	0.663±0.034, 389 (0.685/0.680/0.673/0.661/0.393)	0.626±0.062, 32 (0.684/0.663/0.641/0.617/0.374)	0.598±0.120, 61 (0.682/0.671/0.649/0.588/0.234)	0.584±0.110, 64 (0.682/0.656/0.628/0.574/0.246)	0.644±0.066, 244 (0.725/0.678/0.671/0.639/0.275)	0.667±0.017, 27 (0.683/0.679/0.673/0.663/0.611)	0.628±0.104, 63 (0.682/0.679/0.672/0.658/0.274)	5.80E-22	0.644±0.072, 880 (0.725/0.679/0.670/0.646/0.234/)
**LR score**	0.945±0.061, 389 (0.996/0.982/0.964/0.937/0.495)	0.863±0.103, 32 (0.992/0.947/0.868/0.808/0.470)	0.778±0.235, 61 (0.966/0.915/0.881/0.755/0.110)	0.744±0.215, 64 (0.978/0.882/0.810/0.707/0.075)	0.892±0.127, 244 (0.994/0.970/0.926/0.871/0.149)	0.940±0.036, 27 (0.987/0.978/0.932/0.912/0.871)	0.856±0.201, 63 (0.989/0.956/0.934/0.900/0.153)	6.11E-38	0.895±0.143, 880 (0.996/0.971/0.944/0.879/0.075/)

The varied performances of these methods in different genes and the different scores of each method for different genes suggest that using gene-specific thresholds for deleterious mutations may improve the prediction performance of these *in silico* methods. We screened each gene and identified the gene-specific threshold with maximum MCC. Nonsynonymous single-nucleotide mutations in the diabetes genes from the NHLBI GO Exome Sequencing Project (ESP) [33], [34], the ARIC samples [35] in the CHARGE Exome Sequencing Project [36], and the 1000 Genome Project [37], were used as controls without including mutations recorded in the HGMD database. Shown by our analysis (Table S1), we have been able to improve the prediction performance of each method in most cases, with the FATHMM method as an exception because of its nil/low FNRs in those diabetes genes. For example, the FNR of GERP++ RS for *HNF4A* mutations and the FNR of LRT for *HNF1B* mutations were decreased without any obvious change of their FPRs. On the other hand, redefined thresholds are able to decrease the FPRs of LRT for *INS* mutations, MutationTaster for *ABCC8* mutations, LR score for *INS* mutations, LRT for *ABCC8* mutations, MutationTaster for *INS* mutations, and MutationTaster for *HNF1B* mutations, without obviously increasing the FNRs. The general performances of different methods were summarized in Table 6. From low to high MCCs, the methods were sorted from left to right and from top to bottom. The average difference of MCCs and P value of each two methods was shown.

**Table 6:** Comparisons of the performances of different methods by MCCs [Average difference (P value)].

Methods	FATHMM	LRT	GERP++ RS	SIFT	PhyloP	SiPhy	MutationTaster	Mutation Assessor	LR score	PolyPhen-2 HDIV	PolyPhen-2 HVAR
**LRT**	0.061 (P = 0.018)										
**GERP++ RS**	0.061 (P = 0.346)	0.001 (P = 0.991)									
**SIFT**	0.064 (P = 0.303)	0.004 (P = 0.959)	0.003 (P = 0.963)								
**PhyloP**	0.076 (P = 0.293)	0.015 (P = 0.837)	0.015 (P = 0.452)	0.012 (P = 0.843)							
**SiPhy**	0.113 (P = 0.059)	0.052 (P = 0.362)	0.052 (P = 0.104)	0.049 (P = 0.473)	0.037 (P = 0.382)						
**MutationTaster**	0.117 (P = 0.056)	0.056 (P = 0.317)	0.056 (P = 0.266)	0.053 (P = 0.423)	0.041 (P = 0.497)	0.004 (P = 0.889)					
**Mutation Assessor**	0.153 (P = 0.012)	0.093 (P = 0.106)	0.092 (P = 0.16)	0.089 (P = 0.127)	0.077 (P = 0.267)	0.04 (P = 0.455)	0.036 (P = 0.516)				
**LR score**	0.188 (P = 0.000174)	0.127 (P = 0.013)	0.127 (P = 0.081)	0.124 (P = 0.063)	0.112 (P = 0.143)	0.075 (P = 0.122)	0.071 (P = 0.133)	0.035 (P = 0.404)			
**PolyPhen-2 HDIV**	0.21 (P = 0.00144)	0.149 (P = 0.021)	0.148 (P = 0.00881)	0.145 (P = 0.015)	0.134 (P = 0.019)	0.097 (P = 0.019)	0.093 (P = 0.056)	0.056 (P = 0.158)	0.022 (P = 0.537)		
**PolyPhen-2 HVAR**	0.211 (P = 0.000444)	0.15 (P = 0.0064)	0.15 (P = 0.01)	0.147 (P = 0.037)	0.135 (P = 0.034)	0.098 (P = 0.01)	0.094 (P = 0.026)	0.058 (P = 0.192)	0.023 (P = 0.445)	0.001 (P = 0.934)	
**RadialSVM score**	0.231 (P = 0.0023)	0.17 (P = 0.01)	0.17 (P = 0.033)	0.167 (P = 0.062)	0.155 (P = 0.075)	0.118 (P = 0.05)	0.114 (P = 0.073)	0.078 (P = 0.05)	0.043 (P = 0.341)	0.021 (P = 0.602)	0.02 (P = 0.577)

The varied performance of different methods in different genes is related to specific molecular mechanisms of diabetes mutations. For the 41 *INS* mutations involved in this study, 34 mutations cause neonatal diabetes. These mutations exert diabetic effects by causing misfolding of the insulin protein, rather than inactivating the gene [49], [50]. The dominantly inherited mode of the disease is from dominant negative mechanism, instead of haploinsufficiency. The misfolded insulin protein interferes cellular processes, leading to severe endoplasmic reticulum stress and potentially β cell death by apoptosis [50]. In contrast, a heterozygous individual with one copy of inactivating *INS* mutation may still have a sufficient response to metabolic regulation, thus without neonatal diabetes. For the prediction of neonatal diabetes mutations in the *INS* gene, a protein structure-based prediction method may thus have better performance than others. In this study, we see that PolyPhen-2 with structure-based predictive features has better performance than the more sequence-based SIFT method (Table S1). Unlike other monogenic diabetes genes, the neonatal diabetes mutations in *ABCC8* and *KCNJ11* are gain-of-function mutations [51]. Sequence-based method like SIFT has also lower performance for these mutations than PolyPhen-2.

We acknowledge the current publication bias of diabetes mutations (i.e. the bias towards identifying and reporting diabetes-causing mutations in the general human population). The diabetes mutations have been identified by studies involving much larger number of human individuals, while the genome sequencing projects involved limited number of human subjects. For a disease-causing mutation, no matter its low frequency, as long as the mutation is identified, it will be included. For example, in the case of *GCK* and *HNF1A* genes, the numbers of reported diabetes mutations are much larger than control mutations (479 *vs.* 22, 324 *vs.* 78, respectively). We also want to emphasize the application of gene-specific mutations as functionally neutral controls. Our analysis showed that different methods using redefined thresholds by genome-wide control mutations, instead of gene-specific controls, tend to have poor performances (data available upon request). To acquire a satisfactory MCC tends to need a large number of both diabetes mutations and functional neutral mutations. The gene-specific prediction model proposed by our study will have further improved performance with the availability of sequencing data of a larger number of human individuals.

In conclusion, the available *in silico* methods for the prediction of diabetes mutations have varied performances across different genes. In spite of the high statistical significance of correlations between different methods, different methods may not be able to replace each other. Because of varied performances across genes, applying gene-specific thresholds when possible (i.e. for genes with a number of disease mutations identified and the ROC analysis feasible) may be able to increase the performance of prediction. For genes without sufficient numbers of mutations for the ROC analysis, a consensus threshold should be used [52]. Nevertheless, the limitations of the above methods warrant that new methods are being developed continuously. For example, Johansen et al. recently developed a sequence conservation-based artificial neural network predictor called NetDiseaseSNP [53]. Capriotti et al. developed a Meta-SNP algorithm for the detection of disease-associated nsSNVs, which integrates four different methods: PANTHER, PhD-SNP, SIFT and SNAP. They showed these methods are orthogonal with different biologically relevant relationships, and the integration of different methods achieved higher accuracy [54].

## Supporting Information

Table S1 Method comparisons for gene-specific function prediction for non-synonymous mutations causing diabetes.
(XLS)

## References

[1] Thusberg J, Vihinen M (2009) Pathogenic or not? And if so, then how? Studying the effects of missense mutations using bioinformatics methods. Human mutation 30: 703–714. 19267389 10.1002/humu.20938

[2] Thusberg J, Olatubosun A, Vihinen M (2011) Performance of mutation pathogenicity prediction methods on missense variants. Human mutation 32: 358–368. 21412949 10.1002/humu.21445

[3] Dong C, Wei P, Jian X, Boerwinkle E, Wang K, et al Comparison of functional prediction methods for nonsynonymous SNPs in exome sequencing studies of human diseases. Submitted 10.1093/hmg/ddu733 PMC4375422 25552646

[4] Fajans SS, Bell GI, Polonsky KS (2001) Molecular Mechanisms and Clinical Pathophysiology of Maturity-Onset Diabetes of the Young. N Engl J Med 345: 971–980. 11575290 10.1056/NEJMra002168

[5] American Diabetes A (2007) Standards of Medical Care in Diabetes–2007. Diabetes Care 30: S4–41. 17192377 10.2337/dc07-S004

[6] Odom DT, Zizlsperger N, Gordon DB, Bell GW, Rinaldi NJ, et al (2004) Control of pancreas and liver gene expression by HNF transcription factors. Science 303: 1378–1381. 14988562 10.1126/science.1089769 PMC3012624

[7] Matschinsky FM (1990) Glucokinase as glucose sensor and metabolic signal generator in pancreatic beta-cells and hepatocytes. Diabetes 39: 647–652. 2189759 10.2337/diab.39.6.647

[8] Thomas H, Jaschkowitz K, Bulman M, Frayling TM, Mitchell SM, et al (2001) A distant upstream promoter of the HNF-4alpha gene connects the transcription factors involved in maturity-onset diabetes of the young. Hum Mol Genet 10: 2089–2097. 11590126 10.1093/hmg/10.19.2089

[9] Wang L, Coffinier C, Thomas MK, Gresh L, Eddu G, et al (2004) Selective Deletion of the Hnf1{beta} (MODY5) Gene in {beta}-Cells Leads to Altered Gene Expression and Defective Insulin Release. Endocrinology 145: 3941–3949. 15142986 10.1210/en.2004-0281

[10] Malecki MT, Jhala US, Antonellis A, Fields L, Doria A, et al (1999) Mutations in NEUROD1 are associated with the development of type 2 diabetes mellitus. Nat Genet 23: 323–328. 10545951 10.1038/15500

[11] Neve B, Fernandez-Zapico ME, Ashkenazi-Katalan V, Dina C, Hamid YH, et al (2005) Role of transcription factor KLF11 and its diabetes-associated gene variants in pancreatic beta cell function. Proc Natl Acad Sci U S A 102: 4807–4812. 15774581 10.1073/pnas.0409177102 PMC554843

[12] Raeder H, Johansson S, Holm PI, Haldorsen IS, Mas E, et al (2006) Mutations in the CEL VNTR cause a syndrome of diabetes and pancreatic exocrine dysfunction. Nat Genet 38: 54–62. 16369531 10.1038/ng1708

[13] Plengvidhya N, Kooptiwut S, Songtawee N, Doi A, Furuta H, et al (2007) PAX4 mutations in Thais with maturity onset diabetes of the young. J Clin Endocrinol Metab 92: 2821–2826. 17426099 10.1210/jc.2006-1927

[14] Haneda M, Chan SJ, Kwok SC, Rubenstein AH, Steiner DF (1983) Studies on mutant human insulin genes: identification and sequence analysis of a gene encoding [SerB24]insulin. Proc Natl Acad Sci U S A 80: 6366–6370. 6312455 10.1073/pnas.80.20.6366 PMC394298

[15] Borowiec M, Liew CW, Thompson R, Boonyasrisawat W, Hu J, et al (2009) Mutations at the BLK locus linked to maturity onset diabetes of the young and beta-cell dysfunction. Proc Natl Acad Sci U S A 106: 14460–14465. 19667185 10.1073/pnas.0906474106 PMC2732833

[16] Ashcroft FM, Rorsman P (1989) Electrophysiology of the pancreatic beta-cell. Prog Biophys Mol Biol 54: 87–143. 2484976 10.1016/0079-6107(89)90013-8

[17] Ashcroft FM, Gribble FM (1998) Correlating structure and function in ATP-sensitive K+ channels. Trends Neurosci 21: 288–294. 9683320 10.1016/s0166-2236(98)01225-9

[18] Pearson ER, Liddell WG, Shepherd M, Corrall RJ, Hattersley AT (2000) Sensitivity to sulphonylureas in patients with hepatocyte nuclear factor-1alpha gene mutations: evidence for pharmacogenetics in diabetes. Diabet Med 17: 543–545. 10972586 10.1046/j.1464-5491.2000.00305.x

[19] Frayling TM, Evans JC, Bulman MP, Pearson E, Allen L, et al (2001) beta-cell genes and diabetes: molecular and clinical characterization of mutations in transcription factors. Diabetes 50 Suppl 1: S94–100. 11272211 10.2337/diabetes.50.2007.s94

[20] Feigerlova E, Pruhova S, Dittertova L, Lebl J, Pinterova D, et al (2006) Aetiological heterogeneity of asymptomatic hyperglycaemia in children and adolescents. Eur J Pediatr 165: 446–452. 16602010 10.1007/s00431-006-0106-3

[21] Ellard S, Beards F, Allen LI, Shepherd M, Ballantyne E, et al (2000) A high prevalence of glucokinase mutations in gestational diabetic subjects selected by clinical criteria. Diabetologia 43: 250–253. 10753050 10.1007/s001250050038

[22] Velho G, Froguel P, Gloyn A, Hattersley A (2004) Maturity onset diabetes of the young type 2.

[23] Martin D, Bellanné-Chantelot C, Deschamps I, Froguel P, Robert J-J, et al (2008) Long-Term Follow-Up of Oral Glucose Tolerance Test–Derived Glucose Tolerance and Insulin Secretion and Insulin Sensitivity Indexes in Subjects With Glucokinase Mutations (MODY2). Diabetes Care 31: 1321–1323. 18411240 10.2337/dc07-2017 PMC2453674

[24] Miura A, Yamagata K, Kakei M, Hatakeyama H, Takahashi N, et al (2006) Hepatocyte nuclear factor-4alpha is essential for glucose-stimulated insulin secretion by pancreatic beta-cells. Journal of Biological Chemistry 281: 5246–5257. 16377800 10.1074/jbc.M507496200

[25] Menzel R, Kaisaki PJ, Rjasanowski I, Heinke P, Kerner W, et al (1998) A low renal threshold for glucose in diabetic patients with a mutation in the hepatocyte nuclear factor-1alpha (HNF-1alpha) gene. Diabet Med 15: 816–820. 9796880 10.1002/(SICI)1096-9136(199810)15:10<816::AID-DIA714>3.0.CO;2-P

[26] Shepherd M, Shields B, Ellard S, Rubio-Cabezas O, Hattersley A (2009) A genetic diagnosis of HNF1A diabetes alters treatment and improves glycaemic control in the majority of insulin-treated patients. Diabetic Medicine 26: 437–441. 19388975 10.1111/j.1464-5491.2009.02690.x

[27] Ellard S, Bellanne-Chantelot C, Hattersley AT (2008) Best practice guidelines for the molecular genetic diagnosis of maturity-onset diabetes of the young. Diabetologia 51: 546–553. 18297260 10.1007/s00125-008-0942-y PMC2270360

[28] Qu HQ, Li Q, Lu Y, Fisher-Hoch SP, McCormick JB (2012) Diabetes related DNA mutations in Americans of Mexican Origin with Health Disparities Disclosed by NextGen Sequencing. The American Society of Human Genetics 2012 Annual Meeting Available: http://www.ashg.org/2012meeting/abstracts/fulltext/f120120262.htm. Accessed 24 July 2014.

[29] Shields B, McDonald T, Ellard S, Campbell M, Hyde C, et al (2012) The development and validation of a clinical prediction model to determine the probability of MODY in patients with young-onset diabetes. Diabetologia 55: 1265–1272. 22218698 10.1007/s00125-011-2418-8 PMC3328676

[30] Stenson PD, Ball EV, Mort M, Phillips AD, Shiel JA, et al (2003) Human gene mutation database (HGMD): 2003 update. Human mutation 21: 577–581. 12754702 10.1002/humu.10212

[31] Ng PC, Henikoff S (2003) SIFT: Predicting amino acid changes that affect protein function. Nucleic Acids Res 31: 3812–3814. 12824425 10.1093/nar/gkg509 PMC168916

[32] Adzhubei IA, Schmidt S, Peshkin L, Ramensky VE, Gerasimova A, et al (2010) A method and server for predicting damaging missense mutations. Nat Methods 7: 248–249. 20354512 10.1038/nmeth0410-248 PMC2855889

[33] Tennessen JA, Bigham AW, O'Connor TD, Fu W, Kenny EE, et al (2012) Evolution and functional impact of rare coding variation from deep sequencing of human exomes. Science 337: 64–69. 22604720 10.1126/science.1219240 PMC3708544

[34] Fu W, O'Connor TD, Jun G, Kang HM, Abecasis G, et al (2013) Analysis of 6,515 exomes reveals the recent origin of most human protein-coding variants. Nature 493: 216–220. 23201682 10.1038/nature11690 PMC3676746

[35] The Atherosclerosis Risk in Communities (ARIC) Study: design and objectives. The ARIC investigators. Am J Epidemiol 129: 687–702. 2646917

[36] Morrison AC, Voorman A, Johnson AD, Liu X, Yu J, et al (2013) Whole-genome sequence–based analysis of high-density lipoprotein cholesterol. Nature 201: 3. 10.1038/ng.2671 PMC4030301 23770607

[37] An integrated map of genetic variation from 1,092 human genomes. Nature 491: 56–65. 23128226 10.1038/nature11632 PMC3498066

[38] Pollard KS, Hubisz MJ, Rosenbloom KR, Siepel A (2010) Detection of nonneutral substitution rates on mammalian phylogenies. Genome Res 20: 110–121. 19858363 10.1101/gr.097857.109 PMC2798823

[39] Davydov EV, Goode DL, Sirota M, Cooper GM, Sidow A, et al (2010) Identifying a high fraction of the human genome to be under selective constraint using GERP++. PLoS computational biology 6: e1001025. 21152010 10.1371/journal.pcbi.1001025 PMC2996323

[40] Garber M, Guttman M, Clamp M, Zody MC, Friedman N, et al (2009) Identifying novel constrained elements by exploiting biased substitution patterns. Bioinformatics 25: i54–i62. 19478016 10.1093/bioinformatics/btp190 PMC2687944

[41] Chun S, Fay JC (2009) Identification of deleterious mutations within three human genomes. Genome Research 19: 1553–1561. 19602639 10.1101/gr.092619.109 PMC2752137

[42] Schwarz JM, Rodelsperger C, Schuelke M, Seelow D (2010) MutationTaster evaluates disease-causing potential of sequence alterations. Nat Methods 7: 575–576. 20676075 10.1038/nmeth0810-575

[43] Reva B, Antipin Y, Sander C (2011) Predicting the functional impact of protein mutations: application to cancer genomics. Nucleic Acids Res 39: e118. 21727090 10.1093/nar/gkr407 PMC3177186

[44] Shihab HA, Gough J, Cooper DN, Day IN, Gaunt TR (2013) Predicting the functional consequences of cancer-associated amino acid substitutions. Bioinformatics 29: 1504–1510. 23620363 10.1093/bioinformatics/btt182 PMC3673218

[45] Liu X, Jian X, Boerwinkle E (2011) dbNSFP: a lightweight database of human nonsynonymous SNPs and their functional predictions. Hum Mutat 32: 894–899. 21520341 10.1002/humu.21517 PMC3145015

[46] Liu X, Jian X, Boerwinkle E (2013) dbNSFP v2.0: a database of human non-synonymous SNVs and their functional predictions and annotations. Hum Mutat 34: E2393–2402. 23843252 10.1002/humu.22376 PMC4109890

[47] Wang L-L, Liu Y-H, Meng L-L, Li CG, Zhou S-F (2011) Phenotype Prediction of Non-Synonymous Single-Nucleotide Polymorphisms in Human ATP-Binding Cassette Transporter Genes. Basic & Clinical Pharmacology & Toxicology 108: 94–114. 20849526 10.1111/j.1742-7843.2010.00627.x

[48] Qu HQ, Li Q, Rentfro AR, Fisher-Hoch SP, McCormick JB (2011) The definition of insulin resistance using HOMA-IR for Americans of Mexican descent using machine learning. PLoS One 6: e21041. 21695082 10.1371/journal.pone.0021041 PMC3114864

[49] Colombo C, Porzio O, Liu M, Massa O, Vasta M, et al (2008) Seven mutations in the human insulin gene linked to permanent neonatal/infancy-onset diabetes mellitus. The Journal of clinical investigation 118: 2148. 18451997 10.1172/JCI33777 PMC2350430

[50] Støy J, Edghill EL, Flanagan SE, Ye H, Paz VP, et al (2007) Insulin gene mutations as a cause of permanent neonatal diabetes. Proceedings of the National Academy of Sciences 104: 15040–15044. 10.1073/pnas.0707291104 PMC1986609 17855560

[51] Edghill EL, Flanagan SE, Ellard S (2010) Permanent neonatal diabetes due to activating mutations in ABCC8 and KCNJ11. Reviews in endocrine and metabolic disorders 11: 193–198. 20922570 10.1007/s11154-010-9149-x

[52] Gonzalez-Perez A, Lopez-Bigas N (2011) Improving the assessment of the outcome of nonsynonymous SNVs with a consensus deleteriousness score, Condel. Am J Hum Genet 88: 440–449. 21457909 10.1016/j.ajhg.2011.03.004 PMC3071923

[53] Johansen MB, Izarzugaza JM, Brunak S, Petersen TN, Gupta R (2013) Prediction of disease causing non-synonymous SNPs by the Artificial Neural Network Predictor NetDiseaseSNP. PloS one 8: e68370. 23935863 10.1371/journal.pone.0068370 PMC3723835

[54] Capriotti E, Altman RB, Bromberg Y (2013) Collective judgment predicts disease-associated single nucleotide variants. BMC Genomics 14 Suppl 3: S2. 10.1186/1471-2164-14-S3-S2 PMC3839641 23819846

[55] Blanchette M, Kent WJ, Riemer C, Elnitski L, Smit AF, et al (2004) Aligning multiple genomic sequences with the threaded blockset aligner. Genome Research 14: 708–715. 15060014 10.1101/gr.1933104 PMC383317

